# Supplementary material for: Patterns of Intron Gain and Loss in Fungi
Source: PLoS Biol. 2004 Nov 30;2(12):e422. doi: 10.1371/journal.pbio.0020422 (PMC532390; doi:10.1371/journal.pbio.0020422)
Supplement: Table S1 — Also available at http://genes.mit.edu/NielsenEtAl/. (4.3 MB ZIP). [file pbio.0020422.st001.zip › NielsenEtAl/html/851.html]

AN1168.1.NCU02762.1.MG05643.1.FG01364.1


```
 CLUSTAL W (1.82) Multiple Sequence Alignments - Introns Inserted


Sequence 1: NCU02762.1	2218 aa
Sequence 2: FG01364.1	2103 aa
Sequence 3: MG05643.1	2128 aa
Sequence 4: AN1168.1	2110 aa
Alignment Length: 2263 aa
Number Identitical Residues: 768 aa
Alignment Score (without introns) 40765


MG05643.1 	-----MADPSR----YPQQPPYLNVSEYSQGPEDS----SNGQDGSQQQRGRQPSQAAP-
NCU02762.1	MSDHPAPSPRRASVSSPSHQQSIPLRDLRPVSEEQEGGGGRTSGTDNNVRNSGAGLQMPS
FG01364.1 	-----MSSNSWDQDGRPARRQSIPLQDLDGSHVDN----SQTQAGLFYQQPASSSFDTP-
AN1168.1  	--MASNSHDRGSSDENDIADHSIPLQDLTGPQERG-----RRLSRVGSRLFSGRSLTERG
          	    . .     .         : : :             .             .    .

MG05643.1 	------SGLAP--STYWDNSAHGVRSNEGTGEDFVDDPMALQFALPPDIN-----PRRPS
NCU02762.1	PLAAPMSANLPGLSTYWEHPYAPGHDHRSAAGSLNDH-MALQFALPPNIPGHGHEPLPSA
FG01364.1 	----QMSANAPGISTYWDQPYDHFNDDVSPG-SPIDP-MALQAALPPGMD--HHEPSPPL
AN1168.1  	RPYERLSEDSPVEHGGHTTGSHSAEGANQFNVEDPGAFAAAMSSVGLGLEVSGFQHSQPS
          	      *   *             ..      .  .   *   ::  .:     .   . 

MG05643.1 	STQQYPSSIYEPPQ----PYYNQRPQLHVHTDSIDSDRAPLATNAQPISGNLTPG-HDGL
NCU02762.1	SYMPAASDPYSVPH----PYYEERT----DTDSVESDRVPLKPAAQPIG-RLDAPEGEAV
FG01364.1 	PSTPVR--TYDPPA----PYVEDAP----STDYAESDRVPLTAGVEPISGSLSANNLDSR
AN1168.1  	ASHLAAHTRDESESDMEFVRIDSVDPHEAEHYLSPSEIYPDTTPLTSIRHVQPISGASIT
          	.         .   . .    :.            *:  *  .   .*         .  

MG05643.1 	NRDSFQTVRDMDTS----RDNRMLGADLEN--GQQRNRSFGNSLMPDDGGFRRSRTNSTA
NCU02762.1	PRDSFQTVSDLGSGSSRPRNT-MLGFDLENNFTADRYRSYGGSLVPSDG--RRSRSLSTS
FG01364.1 	NRDSFQTVSSVENSPSRGRSTRSLGEDLRASYGSTRNRNFGASLNPNEY--PRPRSPSTS
AN1168.1  	PSGSQDPSNAPSVRFDDSQMGSRLGDDLHNMESGYSGRRRAESVSKDGG---RSLSPSVS
          	  .* :.        .  :    ** **.        *  . *:  .     *. : *.:

MG05643.1 	SGALSRTASMVRAMSQRVVNISGENEVLEQQARR-RSRSPS---AERRHFADAPIPVDTS
NCU02762.1	-GALSRAGSIMRAMSQRVVMISGEGDLIDQQTQRDRSRSPS-MDRPRTGHASGPMLVDTS
FG01364.1 	-GALSRAGSIVRAMSQRVVNISGETEVIDHRASRHRSRSPVGHSQDRNRDTVNSMFADTS
AN1168.1  	GSALLRASSMMKSVSQRVVNLSNEPEVVERSLSRDSHKSSR-----MEAPPSLPSLTDYA
          	..** *:.*:::::***** :*.* :::::   *   :*.          .  .  .* :

MG05643.1 	YSSQMYPNQEKHPASPNLSEAPPPPTFQQFRRPQPMINPLKGKSLGIFGPENPIRKALCN
NCU02762.1	YPSQMEQNPTEKRPESHHVFPQHPAFRPRSRNP--RNNPLKGKSLGIFSPDNPLRLWLCD
FG01364.1 	YQPQLVRSSSEKSTQPRFGVEETEDPE-LPRPP--LPNPLKGKSLGIFSPNNPLRLRLCD
AN1168.1  	HDAPSSSSLNEQSAREKSVNS-------KAWKG--LSNPLRGKALGFLGPDNALRMWLCD
          	: .    .  :: .  .                    ***:**:**::.*:*.:*  **:

MG05643.1 	ILVLPWTEPLILLLIVAQAVLLAVEAAPTVEE--GGRGDRFG-SAIDWAVLGLFIVFTLE
NCU02762.1	LLVNPWAEPLILILIVLQAVLLAVEAAPDVLADGNGRPERWGSTRIDWAIFVLFVIFTIE
FG01364.1 	LLVNPYTEPFLLLLIVLQAILLAVESAPNVFE--KGRPDRWGKHAIDWAMLALFIIFTLE
AN1168.1  	ILVHPLTEPFILVIIVVQTILLTIESAQSEWS--HPRLPHWGLNRMDYPYFVIFIIYTVE
          	:** * :**::*::** *::**::*:*         *  ::*   :*:. : :*:::*:*

MG05643.1 	VIARIIVSGFILNAAEYSTIDRKRGIKVAVADKYKQVFQPTRQKSIRRPTQAEHYGPSTF
NCU02762.1	LIARIIVSGFIINAPEYEEAKNKRRFRERLAEQYRVIFRPQRQRSVRQTRQEPFE-PAPF
FG01364.1 	IITRIIVSGFILNAPEYSTIDRKRGIRAAIADQYRAVFQPERIKSVKKASQYNPE-PSAI
AN1168.1  	LIAKILVSGLFFNPTEYSTIDRSKGITTALMEKGKNLITPQRQFSIRKSSAQPEPQQASI
          	:*::*:***:::*..**.  ...: :   : :: : :: * *  *:::.        :.:

MG05643.1 	ARSIT-IMQG--QAVPETIEEQQRMQLARRAFLRHGFNRLDFIAVVAFWISFGLSVTGLE
NCU02762.1	TRSFT-MAHS----QIQTLEDQQRRHLARRAFLRHGFNRLDFVAVVSFWISFVLGVTGLE
FG01364.1 	SRSFT-TFMQGQQALPRTLEEHQRFQLARRAFLRHSFNRFDFVAVVSYWITFWLSVSGLE
AN1168.1  	IRTFTGGLNQLEQQLADDPLQKRRIRLAHRAFLRHSFNRLDFVAVLAYWVSFVLNLGGVE
          	 *::*.     ..       :::* :**:******.***:**:**:::*::* *.: *:*

MG05643.1 	KELHLWVFKMLSSLRIVRLLAITKGNA~IILRSLKKSAPLLVRVSFLIGFFWLLFAIIGV
NCU02762.1	SKHHMYVFRMLSCLRIIRLLALTKGNL~IILRSLKKAAPLLVRVSFLMGFFWLLFAIIGV
FG01364.1 	TKHDIYAFRMLSCLRILRLLALTNGTA0IILRSLKKATPLLARVAFLITFFWLLFAIIGV
AN1168.1  	NTHQLYVFRMLSCLRILRLLALTSGTS0VILRSLKKAAPLLVHVAFLIGFFWLLFAIVGI
          	.  .::.*:***.***:****:*.*.  :*******::***.:*:**: ********:*:

MG05643.1 	QSFKGSLKRSCFWVNPEQ-TNNISAGFQN-DDAQCGGWLDN-TGTMKAWIRLPPNETGES
NCU02762.1	QSFKSSLSRQCLWIDPDD-PTNFQAAYTP-DMTFCGGHIDNVTGETMPWVYSHEKES---
FG01364.1 	QSFKASLMRQCVWLDPLE-PTNLDASYTN-SDAFCGGYLKNQTGETMPWLKFNNSES---
AN1168.1  	QSFKSSFRRTCVWLGEEYGEQNFTMNDPFGTLQFCGGWLDE-TGTQRPWYSSDGFES---
          	****.*: * *.*:.   .  *:      .    *** :.: **   .*      *:   

MG05643.1 	GKLLSKPLFDFDVEGSPKGFICPRGSVCLEQDNPHNGTVNFDNILNSLELVFVVMSANTW
NCU02762.1	----LIDLKNGTSRA--KGFICPRGSICIRQENPFNGTVNFDDIGHSLELVFVIMSANTW
FG01364.1 	----LTDLINGTKEG--KGFLCPRGSICLQQSNPYNGTVNFDNIFNSLELVFVIMSANTF
AN1168.1  	-----------DSKP--KGFICPQGSICVEGGNPYNTTMSFDDILHSLELVFVIMSSNTF
          	             .   ***:**:**:*:.  **.* *:.**:* :*******:**:**:

MG05643.1 	SDLMYQMTNTDYLAAALYFAAAIVIMMLWMTNLLIAVITSSFQVIREESKASAFTADQDA
NCU02762.1	SDLMYHTIRSDFLPSALFYGAGILIMLLWMTNLLIAVITSSFQVIREESKASAFTADEPP
FG01364.1 	TDLMYMTMSSDYFQAALFFGAGTMIMMLWLTNLLIAVITSSFQVIREESKASAFTVSEEP
AN1168.1  	TDILYNTTDSDYLVAALFFACGFVILSLWMVNLLVAVITHSFQVIREESKRSAFASQKIE
          	:*::*    :*:: :**::... :*: **:.***:**** ********** ***: .:  

MG05643.1 	YLMNEP--GYPKRVSGLQKIYNKTWWIWPIIIAFDLIAMAWRSVRSSPERVQFIRTTELV
NCU02762.1	GMTSSRVDDTMRRPTLLQRIYNRTKPLWILIIAFGLLAQSLRSARMKSDRERFVNIAEPV
FG01364.1 	-PVQAHSDERVRRTSSLQRVYHKTRAFWILVIAFGLICQACRSATMSSKRARIIDTAEII
AN1168.1  	ASEEEE--ESSRKVSTLKRIYDATYWFWIAVIIFDLVMQAQRSSSMGDGRVQLIDNCEIV
          	   .       :: : *:::*. *  :*  :* *.*:  : **      * :::   * :

MG05643.1 	VTILLVFEIIYRFAADWRDFHRRPRNLVDLFLAIATAVIQIPIIRDSGETYEWLTVFQIL
NCU02762.1	VTLLLDVEIIIRLAADIRGFHRRKRNLVDLGLAVATSIILVPPIRNSGQVYNWLTAFQIL
FG01364.1 	VTILLDIEMIIRIVTDWNYFHKSWRNIFDLGLAIITSIILIPPIRRT-RGYWWLTVFQIL
AN1168.1  	VTVALLGEILLRFISDWRKFHQSRRNWVDLALAIITCIIQVPAIRNSKRTYDILTVFQIL
          	**: *  *:: *: :* . **:  ** .** **: *.:* :* ** : . *  **.****

MG05643.1 	RAYRVVLAIPVTNKLIRLVLGGNASGIGNLLVFVFLMTFLMSIFAAQLFRGELPEQDEDG
NCU02762.1	RVYRLVMVIPMTRKLIRLVLG-NVTGIGNLMSFVFLITFLMAIFAAQLFRGELPREDDGG
FG01364.1 	RVYRIVLAVPVTRELILLVLG-NAAGIGNLMFFVFLMTFLVAIFAAQLFRGQIPLYDDG-
AN1168.1  	RFYRVVLAFAVTRNLITLVFR-NAVGLLNLILFVFLITFLASIFATQLFRSQIPREDDDG
          	* **:*:...:*.:** **:  *. *: **: ****:*** :***:****.::*  *:..

MG05643.1 	EDIKVNFGSIYNSFLGMYQVLTSEDWTDMLYSITGTSQQWGTAWIAGVFFIGWFILSYFI
NCU02762.1	EEIRIPFNTIYNSFLGMYQVMSTENWTDILYSVTASTHHLHTAWIGAIFLVGWFILAYFI
FG01364.1 	ELNRISFYTIYNSFLGMYQILTSENWTGILYTVTSYTRYKNTAWVGAILLIGWFIVAFFI
AN1168.1  	GTIEITFSDLYNSFLGMYIILSSENWTETLYNVTSYTYSFNTAWISATFVILWFIVANFI
          	   .: *  :******** ::::*:**  **.:*. :    ***:.. :.: ***:: **

MG05643.1 	LINMFIAVIQENFDVSEDQKRLEQVKAFLQRKELG--SSSSLTFSRLFTLGK-QPK-KDP
NCU02762.1	LINMFIAVIQENFDVGEDVKRLEQVKAFLQRKELGHSSSSNLALSKVFNFGR-SRKQRDP
FG01364.1 	LVNMFIAVIQENFDVSEDEKRLQQVKAFLQRRDLG-ASSSNLALSTIFGFSK-YRKNKDP
AN1168.1  	VLNMFIAVIQESFDVSEDEKRLHQVRAFLEQKQVGSASQGNLSLSKILKLGRGSARFKDP
          	::*********.***.** ***.**:***:::::* :*...*::* :: :.:.  : :**

MG05643.1 	LDYGPAMMEMLLKEAVVKDFLDDPAVDPLQR-------APTLRDNNLADGPIISGGKFSQ
NCU02762.1	LDYGPAMMEMLLKEAVVHDFLDD-DMDPLQQSSPDGQPTSHLGSSAAMQHDGVTPGVLSS
FG01364.1 	LDYGPATVEMLLKDAVVREFLDD-QMNPKPE------HHGPVRSATTLLGGEIKPGRFSA
AN1168.1  	LDHGPAALDMLLKDAVVREFLDEEMPENQRR------EEASIARTATTAEGLVEPGVFSR
          	**:*** ::****:***::***:   :   .          :          :  * :* 

MG05643.1 	LWQKAVAKMTSRDPNPFYSFTRFNGPNETLDPRKMARDAVQATAARRKAQREYLARHPTY
NCU02762.1	FWGKLVSVFTSREPNPFYSNVNLHGLGENLDPRTMAAQAMNATRRRQRQQREYLMRHPNY
FG01364.1 	VWGKIKDWFSDREPNPFYSSLRFDGANDTSDPRQMAEQAMSATMTRRRAQREYLAKYPNY
AN1168.1  	LWTSVTSSVLRREPNPFYSKLKISRAYEELGPQEMVREVVSAEQQRKREQREYLIRHPTY
          	.* .    .  *:******  .:    :  .*: *. :.:.*   *:: ***** ::*.*

MG05643.1 	NNSMWIFKPSNPIRRLCQRVVGPGRGSERFDGVEPNIYAWYIFSAFIYASILAMVVIACV
NCU02762.1	NNSLFIFKPRNPIRRLCQSLVGPGRGTERFEGAQPNKYAWYTFSTLIYAAIVIMVVLACI
FG01364.1 	NNSLYMFTPNNSLRRLCQRVVGPSRGIERIDGVVPDTVLWYCFTIFVYTAIAAMVILACI
AN1168.1  	NTSLFLFKPDNPVRRLCQRMVGPGRGSQRVEGVDPYKPVWYTFTAVVYAAIVAMVILACI
          	*.*:::*.* *.:***** :***.** :*.:*. *    ** *: .:*::*  **::**:

MG05643.1 	TTPLYQKEYRDRKDFKISNWFVWTDMAFAAIFTTEAFIKVIADGFFFTPNAYFRSSWGLI
NCU02762.1	TTPLYQKEYFEKYGENAFDWFRWVDTGFAIVFTLEALIKVIADGFLWTPNAYFRSTWGVI
FG01364.1 	TTPLYQKEYQEQHDFSIRNWYIWTDMAFAVIFTVEAGIKIIADGLLWTPNAFLRSSWGFM
AN1168.1  	TTPIYQAVNFQTD----RDWFTYTDLGFAILFSIEALIKVIADGFFWTPNAYFRGSWGFI
          	***:**    :       :*: :.* .** :*: ** **:****:::****::*.:**.:

MG05643.1 	DAVVLTTLWISVITVLKNEGSISRAVGAFKALRALRLLNVSDTARNTFHSLIIVQGWKIL
NCU02762.1	DAIVLITLWINVITLFTRNGAVSRAVGAFKALRALRLLNISESAMDTFHSLLIVGGWKIL
FG01364.1 	DSIVLVTLWINVVTLLINDGAISRAIGAFKALRALRLLNVSNSARNTFHDLIIVGWWKIL
AN1168.1  	DGIVLVTLWINVLSALSSERGVSRAIGAFKALRALRLLNFSDSAKNTFHSVIIVGGWKVI
          	*.:** ****.*:: :  : .:***:*************.*::* :***.::**  **::

MG05643.1 	S~AAFVSLSLLIPFALYGLNLFQGQFDECNDGDD-ITLLKDCWGEYMNAPIHDGHSILAP
NCU02762.1	S~AAFVSISLLIPFAIYGVNLFHGKLLACNDSSEGIVSLDDCFGEWNSTPYNNNWPVLAP
FG01364.1 	G0AAFVSLSLLIPAAIYGLNLFNGLLLSCNDGSDGINTMDNCFGEFASTPFSDDWPMLAP
AN1168.1  	A0AALVSMSFLIPFAIYGVTLFNGQLMECN-DDGIAGSLDACVGEFMSSPFN--WDVLAP
          	. **:**:*:*** *:**:.**:* :  ** ..     :. * **: .:*      :***

MG05643.1 	RAVNNPQYSFDDFGSSLFILYQIVSQERWVDVSFSAQAISGIGNQP---IFGNQRGNGMF
NCU02762.1	RSVANPYFSFDDFGSSLFILFQIVSQEGWVDVSFALQAITGRGLQPQSGAPYSGQGNALF
FG01364.1 	RVAANPFYSFDDFGSSLFILFQIVSQEGWTDVSFAVQAITGPFKQP---SDLASQGNAVF
AN1168.1  	RAVSNPWYDFDNFGNSLFILFQIVSQEGWTAVQSSVMSITGLGRQP---ETFAAPENGLF
          	* . ** :.**:**.*****:****** *. *. :  :*:*   **          *.:*

MG05643.1 	FVIFNILSTVFVLTLFISVFMRNYTEQTGVAYLTAEQRSWLELRKLLRQVSPSKTSYDES
NCU02762.1	LVVFNLLATVFVLTLFISVFMRNYTEQTGVAFLTAEQRSWLELRKLLRQISPSKSSYDES
FG01364.1 	IVIFNLLATVFVLTLFISVFMRNYTEQTGVAYLTAEQRSWLELRKILRQISPSKSSYDDS
AN1168.1  	FVVFNLLGAVFVLTLFTSVFMRNYTEQTGVAFLTAEQRSWLELRKLLRQVSPSKRRSSEK
          	:*:**:*.:******* **************:*************:***:****   .:.

MG05643.1 	RTKWKKWCHKRAIEKRGKWYQTVTAVLCLHLALLITEFIDEPDVWSKAREFVFLAFNLVY
NCU02762.1	KNKWKIWCHKRAIEKRGKWYMAITSVLVLHLILLLSEFYSEPDWWTNTRDALFLLFTLIY
FG01364.1 	EKSWKKWCHKRAIEKKGKWYRTITVILVLHLIVLVSEFADEPWWWTRYRDFVFFAFIMAY
AN1168.1  	STSWKQWAYRVAYKKHGRWAKCVTTVLVLHLILLVIEYYPENYLWNLARDGLFFLFNFVY
          	 ..** *.:: * :*:*:*   :* :* *** :*: *:  *   *.  *: :*: * : *

MG05643.1 	LTNIAIRITGLGWTRFRRSSWDMFSLVSVSGTIVTS-VLHLSDPAREVFTQLHKVFLVSI
NCU02762.1	IANIVIRIVGLGWSRFRKSSWDMFSLITVTGAFATT-ILFMTNTKLDTYIQLHKFFLVAI
FG01364.1 	TVNIAIRIIGLGWVRFRRSSWDLYSLFIVFGAFVSTFALLVSDTELEAYVQLHKVFLVAI
AN1168.1  	IANVLLRLVGLGWHRFSTSSWDMYALLVVPSTVITSILNFTSYEQRKTIEVLNKLFLVAI
          	 .*: :*: **** **  ****:::*. * .:. ::     :    ..   *:*.***:*

MG05643.1 	LLLLIPRNGALDQLFKTAAASLTTIGNLVATWFVFYIMFAIALTQSFSLTRFGPEENNNQ
NCU02762.1	VLMLIPRNDALDQLFKTAAASLTTIGSLLATWLVFFLVFAIALTQTFSLTRFGNSTDYYV
FG01364.1 	VLLLIPRNDALDQLFKTAAASLTVIGNLLATWLVFFLVFAIAMTQAFSLTRFGAETSANV
AN1168.1  	TLLLIPRNNQLDQLFKTAAASFTSIGNLLATWFVMFLVYAIAMNQAFGLTKFGGNENGNI
          	 *:*****. ***********:* **.*:***:*:::::***:.*:*.**:** . .   

MG05643.1 	NFRTVPKALILLFRMSAGEGWNTIMEDYAEIEPPMCVES-TNFLNSDCGSKSWARCLFIA
NCU02762.1	NLRTVPNALILLFRFSCGEGWNQVMEDFAKIKPPLCVES-TNFSDSDCGSTAWARALFIA
FG01364.1 	NFRTVPKALILLYRMSLGEGWNAVMEDYASIEPPLCVIDEGNFFNSDCGSKSWARILFIA
AN1168.1  	NFRDIPRALILLFRTSCGEGWNELMEDFATMEPPLCTAITDGFFGNDCGSKGWARALFIS
          	*:* :*.*****:* * ***** :***:* ::**:*.    .* ..****..*** ***:

MG05643.1 	WNIISMYIFVNLFVSLIYESFSYVYQRSSGVDMVDRDEIRRFKEAWRSIDPAGTGFISKE
NCU02762.1	WNIISMYIFVNLFVSLIYESFSYVYQRTSGLAMVDRDELRRFKEAWRSVDPSGTGFISKE
FG01364.1 	WNIVSMYIFVNLFVSLIYESFSYVFQRSSGMAVVDRDEIRRFKEAWRSVDPAGTGYITKA
AN1168.1  	WNIISMYIFVSLFVSLIFESFSYVYQRSSGLYVISREELRRFKQAWAEFDPEGTGYITKE
          	***:******.******:******:**:**: ::.*:*:****:** ..** ***:*:* 

MG05643.1 	AFPRLLG~ELSGVFEMRIYDSEDSVSRILEDCQTNGAAVGRHASMVSQSAYSG-VDLAKL
NCU02762.1	AFPRLLG~ELSGVFEMRIYESHDSVRQILEDVRGEDTSSIRHASIISTTQFQTSLDLKKL
FG01364.1 	AFPRLLG~ELSGVFQMRIYDPEDSVHSILEDIR-DDVKLTHHSSIATTSGFSG-IDLNKL
AN1168.1  	QFPRLLR0ELTGIFEVRIYQGDFMVPRILDECR-----VDKRDSLLAHRRVVEGVDLDKL
          	 *****  **:*:*::***: .  *  **:: :       :: *: :      .:** **

MG05643.1 	NERLARLDVAKVRERRRRYNMFFEEVMVLADPDKGIAFSTVLMILAHYNIISDSKSLR~L
NCU02762.1	NERISQIDVAKVRERRRRFNIFFEEVMVSADPERGISFTTVLMILAHYKIINDSKSLR2L
FG01364.1 	NARLRRIDSEKVRHQRRRFNTFYEEVLVSADPDRGISFTSVLMILAHYNIISDNKSLR2L
AN1168.1  	AKIIRKIPVNTVRERRKRLNELYEEVLVSADPRYGITFHSCLMILAHYNVISDSKSLR~L
          	   : ::   .**.:*:* * ::***:* ***  **:* : *******::*.*.**** *

MG05643.1 	EEFLRRRARLQRVEEEVRRRVVLGFFDTL2FWSRNFKRHLAWKRSGRMTDIPNLDVPEIF
NCU02762.1	EEFLRRRSRLQRVEEEIHRRVVLGFFDMI~YYSRKFQRHMELKRSARLTAIPQLGVPEIY
FG01364.1 	EEFLRRRARLQRVDDQVRRGVVLGFFDTL2YYTRQFKKHLMRKQSARMTAVPQLNIPEIM
AN1168.1  	EEFLRRRAKLQRVEEAVRRNTVIGFFKTL~TATREFRRKIEHKRSARMTFVPQFNVPEIY
          	*******::****:: ::* .*:***. :   :*:*::::  *:*.*:* :*::.:*** 

MG05643.1 	VDDEGGVSPRTRVHKSLPIPGTPSSPRSPFPAHNRDSPFLSP------------------
NCU02762.1	VDNDHENQTDNEQQNKDSNSKNSARASGSGSSTAAGTNFFESTLTNPFGSGSSTVNPGGG
FG01364.1 	VENDLS-----EDETGRAESRSPLSPASRPSSVDAGE-----------------------
AN1168.1  	VDDELQ-EDQQSQDGPLGSARSSGAEPSMLSPTSTGRGEAGP------------------
          	*:::   .     .     . ..    .  ..   .     .                  

MG05643.1 	-------DHLSNHQRKWSAASADMASYSPELSGPHPLSFPRAGQAASGHQRNNPSAFSFD
NCU02762.1	ASFLSADDARTAHHRSWSGASADLSSFDTSYG--HPLAGPRMNKSSNSVSHRNTHAYSFE
FG01364.1 	-------------PRNWLG-SVDLSLHDTSWS--HPLSFPRAAPPSPTVPTQPS-AFSFE
AN1168.1  	----------SQLPRINTNVGDRMNSEASSPSEWSNISISLSPGRERAHTTSSYDPPSFS
          	          :   *     .  :    .. .    :: .               . **.

MG05643.1 	LQQP---------------------------------------PGSNSSSRRGSTLSPTQ
NCU02762.1	LHDSSGIGGGNAPSPGGWGGGSGTMDSMGLGGGSALDDNNGGRSPGGEGTKRGSSVSPSK
FG01364.1 	IEED------EPQQPTAQARTS---------------------APTGRESNHLDPTSPLQ
AN1168.1  	EGAS----------------------------------------ATSEHSRHHSAMAVQD
          	                                              .  :.: .. :  .

MG05643.1 	ARELLDDSVWVESIRRSTTVRQSAWGSRSGSYSPSGAGGGNSRGDGYF
NCU02762.1	LSSMLDDSVWVASIRRSATMRRSQNDTDWGGGSRASGGGYSGAY----
FG01364.1 	VRGMLDDSVWGESIRRSATVRQTQNRS---------------------
AN1168.1  	VMQSLGDSAWGESIRRSFTQRRPRERPRDET-----------------
          	    *.**.*  ***** * *:.   .
```
